# Astaxanthin protects against hearing impairment in diabetic rats

**DOI:** 10.1016/j.bjorl.2022.02.004

**Published:** 2022-02-28

**Authors:** Serdar Ferit Toprak, Serkan Dedeoğlu

**Affiliations:** a Dicle University Medical Faculty, Department of Otorhinolaryngology, Diyarbakir, Turkey; b Gazi Yaşargil Training and Research Hospital, Department of Otorhinolaryngology, Diyarbakir, Turkey

**Keywords:** Diabetes mellitus, Astaxanthin, Hearing loss, Pro-inflammatory cytokines

## Abstract

**Objective:**

Diabetes Mellitus (DM) causes an increase in oxidative stress that leads to deterioration in auditory functions. Astaxanthine (AST) is known to have strong antioxidant effects. In this study, the aim is to investigate the effect of AST against hearing loss that is due to DM.

**Methods:**

This study is an experimental animal study. The study was designed in four groups with 8 animals (n = 8) in each group. The groups were as follows; Control Group (CNT), Diabetic Group (DM), AST applied diabetic group (DM+AST), and AST applied non-diabetic group (AST). Streptozotocin was applied in rats to induce DM. AST was administered by oral gavage. Auditory Brainstem Responses (ABR) and Distortion Product Otoacoustic Emissions (DPOAE) tests were performed on several days of the study. At the end of the study, pro-inflammatory cytokine levels were analyzed in cochlear tissue samples, and Glutathione Peroxidase (GPx), Superoxide Dismutase (SOD), Catalase (CAT) and Malondialdehyde (MDA) levels were measured.

**Results:**

When the findings obtained in the ABR and DPOAE tests in the DM group, it was observed that there was a significant deterioration in the hearing sense. This deterioration was not observed in the DM+AST group. In the DM group, GPx, SOD and CAT levels decreased and MDA levels increased in blood and cochlear tissue. Compared to the DM group, it was noted that antioxidant enzyme levels increased and MDA levels decreased in the DM+AST group. Cochlear tissue pro-inflammatory cytokine levels, which increased with DM, were significantly decreased in the DM+AST group.

**Conclusion:**

Even though the effects of AST were investigated in a diabetic experimental animal model, if this molecule is proven to be effective in diabetic humans, it can be considered an adjunct therapeutic option with its antioxidant effects.

**Level of evidence:**

The level of evidence of this article is 5. This article is an experimental animal and laboratory study.

## Highlights

• Diabetes mellitus causes an increase in oxidative stress that leads to deterioration in auditory functions.
• Astaxanthine is known to have strong antioxidant effects.
• The results show that Astaxanthine has protective effects against hearing impairment due to DM with its antioxidant and anti-inflammatory properties.

## Introduction

Diabetes Mellitus (DM) is a very common chronic metabolic disease characterized by the absence or ineffectiveness of insulin. According to the International Diabetes Federation, nearly 500 million people in the world have this disease. The number of patients with DM is estimated to approach 600 million in 2030. The money spent on DM and its related diseases is over 750 billion dollars and this amount is continually increasing.^1^ One of the reasons why DM is such an important health problem is the serious complications it has caused over the years. As a result of these complications, many organs or functions are adversely affected, especially the kidneys, liver, nervous system and cardiovascular system.^2^, ^3^, ^4^

One of the functions negatively affected by DM is the sense of hearing. It has been reported that sudden or gradual hearing loss can develop at varying rates in DM.^4^, ^5^ According to researches, DM causes hearing loss by affecting the cochlea, vestibulocochlear nerve and the temporal bone.^6^, ^7^ Oxidative stress and inflammation are the mechanisms held responsible here. In DM patients, hyperglycemia leads to an increase in the polyol pathway, aldose reductase activity and advanced glycation end-products production. As a result, increased inflammation and Reactive Oxygen Species (ROS) cause vascular endothelial damage, neuropathy in the vestibulocochlear nerve, and loss of outer vibratile hair cells.^6^, ^8^, ^9^, ^10^ In many studies based on these data, the effect of antioxidant molecules on hearing loss due to DM has been tested and successful results have been reported.^6^, ^9^, ^11^

Astaxanthin (AST) is a member of the carotenoid family, whose well-known members are vitamin C and vitamin E. AST is a molecule that is not synthesized in the human body, and it must be taken from the outside. AST can be found in some natural sources otherwise it can be synthesized chemically in the laboratory environment.^1^ Sea creatures are the most important natural sources of AST. The United States Food and Drug Administration (FDA) approved AST as a nutritional supplement in 1999.^12^ In fact, studies have reported that AST can exert an antioxidant effect up to 100 times more than other carotenoids.^13^ This superiority is due to its molecular structure. AST has effective ROS scavenging effects, especially singlet oxygen. Studies have shown that AST effectively reduces oxidative damage, lipid peroxidation, advanced glycation end-products development and has strong antioxidant effects.^13^, ^14^, ^15^, ^16^

In this study, the aim was to investigate the effects of AST, which has strong antioxidant effects, on hearing loss due to DM. For this purpose, an experimental streptozotocin-induced DM model was conducted. Following the development of DM, rodents were applied with AST, and its protective effects against hearing loss were investigated. In order to reveal the effects of AST, biochemical parameters that may be indicators of oxidation and inflammation, as well as hearing tests were used.

## Materials and methods

### Animals

This study was carried out with the permission of the Experimental Animals Local Ethics Committee (Approval Number: 2021/2). The principles of the ‘Protection of Animal Rights’ determined by the NIH regarding animal rights were carefully followed during the study. In the experimental procedures, 32 male Wistar albino rats weighing approximately 200–250-grams were used. Throughout the experiments, rats were housed in an environment with 12 hours of light/12 hours of darkness at a room temperature of 23 ± 2 °C and a humidity of 60%. The rats were fed ad libitum.

### Experimental procedures

**Figure 1:**
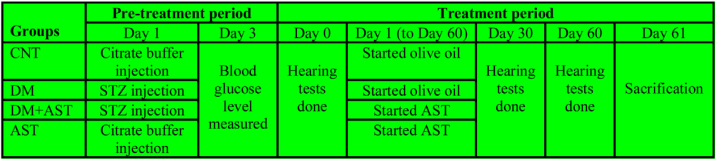
Timeline of experimental procedures. DM was induced in pre-treatment period CNT, Control;, DM, Diabetes Mellitus; AST, Astaxanthin.

The study was designed in four groups with 8 animals (n = 8) in each group. The groups were as follows; Control Group (CNT), diabetic group (DM), AST applied diabetic group (DM + AST), and AST applied non-diabetic group (AST). The experiment was designed in two episodes, and the detailed procedure is shown in Fig. 1.

The first episode was the pre-treatment period in which the diabetes was formed, and the second was the treatment period in which AST was applied to DM rats. The pre-treatment period has lasted for three days, and in this period, DM and DM+AST groups were made diabetic. DM was developed by administration i.p. at a 60 mg/kg dose of Streptozotocin (STZ) dissolved in citrate buffer solution (pH = 4.5). In the same period, the rats not selected for diabetic groups (CNT and AST) were applied with citrate buffer solution to standardize the procedure. Peripheral blood glucose levels were measured by a glucometer in blood samples taken from the tail vein three days after STZ administration. Rats with blood glucose levels above 300 mg/dLwere considered DM.

Before the start of the treatment episode (Treatment period-Day 0), all rats were anesthetized with 50 mg/kg ketamine (Ketalar; Pfizer, Istanbul, Turkey) and 8 mg/kg xylazine hydrochloride (Rompun; Bayer, Istanbul, Turkey) via the intramuscular route. The external ear canal and tympanic membranes of the rats under anesthesia were estimated by otoscopy at the start of the study. The study was continued with the subjects that were found to be normal.

Furthermore, Auditory Brainstem Responses (ABR) and Distortion Product Otoacoustic Emissions (DPOAE) were measured in the anesthetized rats. ABR and DPOAE tests were re-applied to the rats on the 30th and 60thdays of the experiment. After conducting these tests on the rats, the treatment episode was started. The rats in the DM+AST and AST groups were administered AST at a dose of 80 mg/kg once a day for 60 days, in a volume not exceeding 1 mL, by oral gavage. AST was dissolved in olive oil, and its dose was decided by considering similar studies.^14^ Rats in the CNT and DM groups were administered 1 mL of olive oil by oral gavage once a day for 60 days. The drugs were administered at the same time in the morning every day. STZ and AST were obtained from Sigma Chemical (Sigma Chemical Co, St.Louis, MO, USA). During the study, the subjects’ blood glucose levels and body weights were measured at regular intervals. After hearing tests performed on the 60th day of the study, the rats were sacrificed by drawing large amounts of blood. The blood samples were centrifuged, and the supernatant part was separated. The cochleas were removed from the temporal bones. The temporal bones containing the tympanic bullae were cleared off the other structures to reveal the cochleae. Blood and cochlea tissue samples were stored at −80 °C for biochemical analysis.

### ABR measurement

ABR threshold measurement was performed in anesthetized rats in a silent environment. Measurements were made with the Interacoustics EP25 (Interacoustics, Minnesota, USA) device using disposable surface snap electrodes. Electrodes were placed on the vertex, glabella and mastoid areas. The filter was adjusted in the band range of 30‒1500 Hz, with a repetition rate of 21.1 seconds. The stimuli were started at 80 dB and decreased by 10 dB each time. At least two measurements were made to confirm the threshold value.

### DPOAE measurement

Evaluating the peripheral hearing system of rats the DPOAE test was performed using the ILO-288 otoacoustic Emission Instrument (Otodynamics, London, UK). Measurements were made in a special quiet room while the rats were under anesthesia. Neonatal rubber probes were used for measurements. Otoacoustic emissions were measured using different frequencies and intensities. Equilevel primary tones f1 (65 SPL) and f2 (55 SPL) were fixed at f2/f1 = 1.22, and the DPOAE were measured at 8 different frequencies between 1000 and 8000 Hz (1000, 2000, 3000, 4000, 5000, 6000, 7000 and 8000 Hz). The DPOAEs were established in DP-grams.

### Biochemical analyses

ELISA method was used for measuring Glutathione Peroxidase (GPx), Superoxide Dismutase (SOD) and Malondialdehyde (MDA) levels in serum and cochlea tissue samples. The spectrophotometric method was preferred for the measurement of Catalase (CAT) level. Tissues were homogenized by adding 9 times their weight in iced phosphate-buffered saline (PBS; 0.01 M, Ph = 7.4) solution in the analysis of tissue samples. Centrifugation was done at 5000 g for 5 minutes and the supernatant was separated for analysis. Commercial kits (Elabscience, Wuhan, China) were used for the measurement of GPx, SOD, MDA and CAT levels. GPx, MDA and SOD levels were performed by a Robonic readwell touch (Thane, India) automated ELISA plate analyzer. In the spectrophotometric (uv-1205, Shimadzu, Japan) measurement of the CAT level, the wavelength was set to 405 nm.

### Real-time PCR analyses

A real-time PCR device was used to measure expression levels of pro-inflammatory cytokines IL-1β, IL-6 and TNF-α. For the isolation of total RNA from tissue, the ready kit (RNA Easy kit, Qiagen, Germany) was used as described by the manufacturer. Extracted RNA was dissolved in nuclease-free distilled water and stored at −20 °C. Real-time PCR was performed using 2 µL template in a 20-µL reaction containing 0.25 µM of each primer and 12.5 µL Sybr Green Real-time PCR MasterMix (Qiagen, Germany). Each run of 50 °C for 2 min and 95 °C for 10 min followed by 45 cycles of 95 °C for 15 s, 60 °C for 20 s, and 72 °C for 60 s in a real-time qPCR machine (Rotor Gene Q, Qiagen, Germany).

### Statistical analyses

The Kolmogorov-Smirnov test was used to control the conformity of the data obtained in the study to the normal distribution. Normally distributed data were expressed as arithmetic mean ± standard deviation. For the analysis of the data, one-way ANOVA and post hoc Tukey test were used. Repeated measures ANOVA and post-hoc Bonferroni test were used in the analysis of repeated measures. For statistical analysis, SPSS 21.0 program (IBM, Chicago, Il, USA) was used. The significance level was taken as *p* < 0.05.

## Results

### Body weight and blood glucose level results

The body weights and blood glucose levels of the subjects were measured on days 0, 30 and 60. The blood glucose levels in the DM groups were found to be significantly higher when compared to the CNT and AST groups in all three different measurements (*p* < 0.001). In terms of body weights, there was no significant difference between the groups in the measurement made on day 0, while the body weights of the groups with DM were found to be significantly lower in the 30 and 60 day measurements compared to the non-diabetic groups (*p* < 0.001).

### ABR results

ABR thresholds values obtained from rats at day 0, 30 and 60 are shown in Table 1. In the examination of the intergroup comparison results, no significant difference was found between the four groups in the measurements made on day 0 (*p* > 0.05). When the results of day 30 and day 60 were examined, it was observed that the values obtained in the DM group were significantly higher than the values obtained in the other three groups (*p* < 0.05).

**Table 1:** Comparison of ABR thresholds within groups and between groups. Data are shown as mean ± standard deviation.

Groups	Day 0	Day 30	Day 60	Repeated (ANOVA)	Bonferroni test
1 (CNT)	26.2 ± 0.97	26.5 ± 1.41	25.8 ± 2.16	*p* = 0.597	For DM group: D0 vs. D30 (*p* = 0.001); D0 vs. D60 (*p* = 0); D30 vs. D60 (*p* = 0.002)
2 (DM)	26.4 ± 2.26	35.8 ± 3.27	44.7 ± 3.15	***p* = 0**	
3 (DM + AST)	26.7 ± 1.90	27.7 ± 2.91	29.0 ± 2.20	*p* = 0.174	
4 (AST)	26.5 ± 1.69	24.5 ± 1.77	26.0 ± 2.50	*p* = 0.179	
ANOVA (between groups)	*p* = 0.965	***p*** = **0**	***p*** = **0**		
Post hoc Tukey test		For day 30 and 60: DM vs. CNT (*p* = 0); DM vs. DM + AST (*p* = 0); DM vs. AST (*p* = 0)			

CNT, Control; DM, Diabetes Mellitus; AST, Astaxanthin.

No difference was found between the repeated measures in the CNT, DM+AST and AST groups (*p* > 0.05) in the analysis of the measurement results performed on the 0, 30 and 60 days of the same group with the repeated measures ANOVA test. In the DM group, the values reached on the 30th day were found to be significantly higher than on the 0th day, while the values obtained on the 60th day were significantly higher than both 0 and 30 days (*p* = 0 and *p* = 0.002, respectively).

### DPOAE results

**Figure 2:**
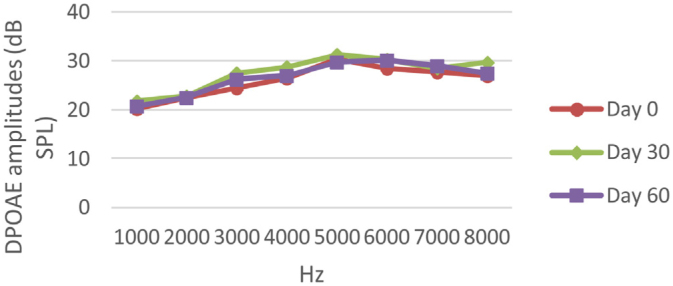
DPOAE results measured at day 0, 30 and 60 of the control group. According to the repeated measures ANOVA test for each frequency, there was no difference between the measurements made at 0, 30 and 60 days. (*p* > 0.05). DPOAE, Distortion Product Otoacoustic Emissions.

**Figure 3:**
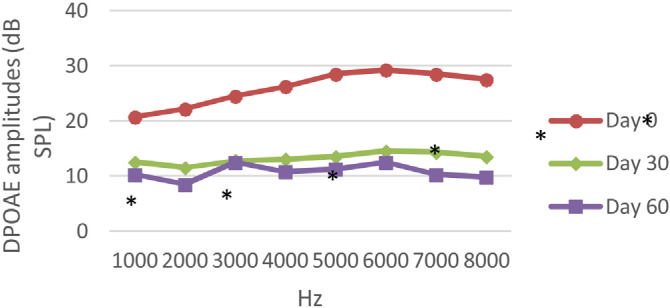
DPOAE results obtained at day 0, 30 and 60 in the DM group. The valuesmeasured on the 30th and 60th days for all frequencies were found to be significantly lower than the values measured on the 0th day. *p < 0.01 for Day 0 vs. Day 30 and Day 60 (repeated measures ANOVA test). DM, Diabetes Mellitus; DPOAE, Distortion Product Otoacoustic Emissions.

**Figure 4:**
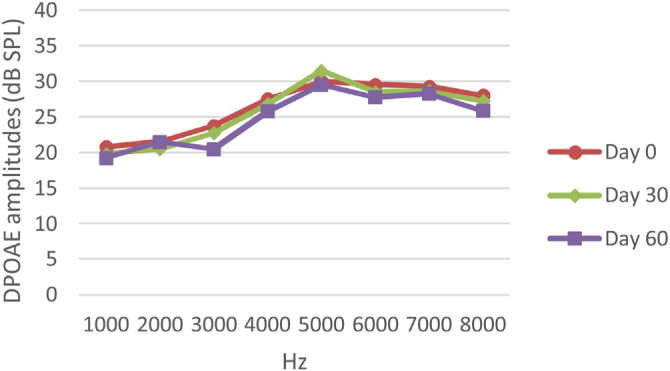
DPOAE results obtained at 0, 30 and 60 days for the DM + AST group. According to the repeated measures ANOVA test for each frequency, no difference was observed between the measurements made at 0, 30 and 60 days (*p* > 0.05). DM, Diabetes mellitus, AST: Astaxanthin, DPOAE, Distortion Product Otoacoustic Emissions.

**Figure 5:**
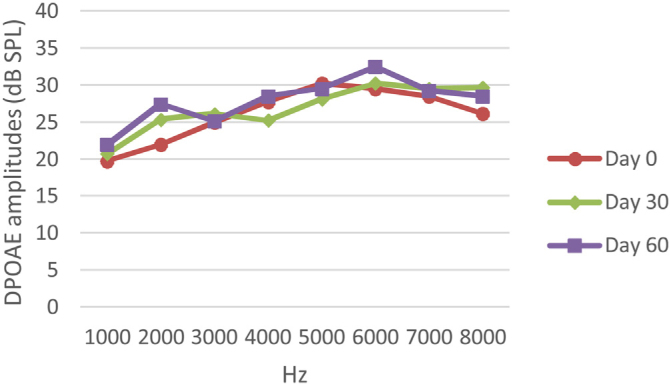
DPOAE results obtained on day 0, 30 and 60 in the AST group. According to the repeated measures ANOVA test for each frequency, there was no difference between the data in the measurements made on the 0, 30 and 60 days (*p* > 0.05). AST, Astaxanthin; DPOAE, Distortion Product Otoacoustic Emissions.

At 0, 30 and 60 days, the DPOAE amplitude values of the subjects were measured. For each amplitude value measured in the CNT, DM+AST and AST groups, no difference was found in repeated measurements performed on days 0, 30 and 60 (*p* > 0.05, Figure 2, Figure 4, Figure 5). In the DM group, the values reached at all frequencies in the measurements made on the 30th and 60th days were found to be significantly lower than the values measured on the 0th day (*p* < 0.01, Fig. 3).

### Biochemical results

Analysis results of serum samples of GPx, SOD, CAT and MDA are shown in Table 2(a), and analysis results of cochlea tissue samples are shown in Table 2(b) in the study. In view of the results obtained from serum and cochlea, Gpx, SOD and CAT levels were found to be significantly lower in the DM group compared to the CNT group, and MDA levels were found to be significantly higher (*p* =  0.001 for cochlea SOD level, *p* =  0 for other results). When the DM+AST group was compared with the DM group, it was observed that GPx, SOD and CAT levels increased significantly in both serum and cochlea results, while MDA levels decreased significantly (respectively for serum *p* = 0.029, *p* = 0.012, *p* = 0.035, *p* = 0.003, *p* = 0.020 for cochlea, *p* = 0.010, *p* = 0.030, *p* = 0). In the AST group only, the results were close to the CNT group.

**Table 2:** GPx, SOD, CAT and MDA results of (a) serum and (b) cochlea tissue samples. Data are presented as mean ± standard deviation.

(a) Results of serum samples				
Groups	GPx (ng/mL)	SOD (ng/mL)	CAT (U/mL)	MDA (ng/mL)
1 (CNT)	12.7 ± 1.98	23.1 ± 1.66	10.8 ± 3.48	1.55 ± 0.87
2 (DM)	3.12 ± 1.45	7.12 ± 3.39	3.05 ± 2.09	5.00 ± 2.28
3 (DM + AST)	7.01 ± 2.26	14.1 ± 3.04	7.87 ± 3.90	2.39 ± 1.01
4 (AST)	15.24 ± 4.12	28.8 ± 4.08	11.87 ± 3.52	1.21 ± 0.53
ANOVA	*p* = 0	*p* = 0	*p* = 0	*p* = 0
Post hoc Tukey test	Gr1 vs. Gr 2 (*p* = 0)	Gr1 vs. Gr 2 (*p* = 0)	Gr1 vs. Gr 2 (*p* = 0)	Gr1 vs. Gr 2 (*p* = 0)
	Gr1 vs. Gr3 (*p* = 0.001)	Gr1 vs. Gr3 (*p* = 0.001)	Gr2 vs. Gr3 (*p* = 0.035)	Gr2 vs. Gr3 (*p* = 0.003)
	Gr2 vs. Gr3 (*p* = 0.029)	Gr1 vs. Gr4 (*p* = 0.047)	Gr2 vs. Gr4 (*p* = 0)	Gr2 vs. Gr4 (*p* = 0)
	Gr2 vs. Gr4 (*p* = 0)	Gr2 vs. Gr3 (*p* = 0.012)		
	Gr3 vs. Gr4 (*p* = 0)	Gr2 vs. Gr4 (*p* = 0)		
		Gr3 vs. Gr4 (*p* = 0)		

(b) Results of cochlea tissue samples				
Groups	GPx (ng/g tissue)	SOD (ng/g tissue)	CAT (U/mg protein)	MDA (ng/g tissue)
1 (CNT)	6.94 ± 2.48	17.9 ± 4.07	7.31 ± 2.85	1.89 ± 0.97
2 (DM)	1.40 ± 0.83	7.49 ± 4.55	1.95 ± 1.03	4.27 ± 1.33
3 (DM + AST)	4.22 ± 1.74	15.6 ± 4.91	5.18 ± 2.10	1.99 ± 0.83
4 (AST)	7.16 ± 1.74	14.5 ± 5.42	7.21 ± 2.33	1.49 ± 0.73
ANOVA	*p* = 0	*p* = 0.001	*p* = 0	*p* = 0
Post hoc Tukey test	Gr1 vs. Gr 2 (*p* = 0)	Gr1 vs. Gr 2 (*p* = 0.001)	Gr1 vs. Gr 2 (p = 0)	Gr1 vs. Gr 2 (*p* = 0)
	Gr1 vs. Gr3 (*p* = 0.026)	Gr2 vs. Gr3 (*p* = 0.010)	Gr2 vs. Gr3 (*p* = 0.030)	Gr2 vs. Gr3 (*p* = 0)
	Gr2 vs. Gr3 (*p* = 0.020)	Gr2 vs. Gr4 (*p* = 0.029)	Gr2 vs. Gr4 (*p* = 0)	Gr2 vs. Gr4 (*p* = 0)
	Gr2 vs. Gr4 (*p* = 0.003)		Gr3 vs. Gr4 (*p* = 0.046)	
	Gr3 vs. Gr4 (*p* = 0.015)			

CNT, Control; DM, Diabetes Mellitus; AST, Astaxanthin; GPx, Glutathione Peroxidase; SOD, Superoxide Dismutase; CAT, Catalase; MDA, Malondialdehyde.

### Real time PCR results

**Figure 6:**
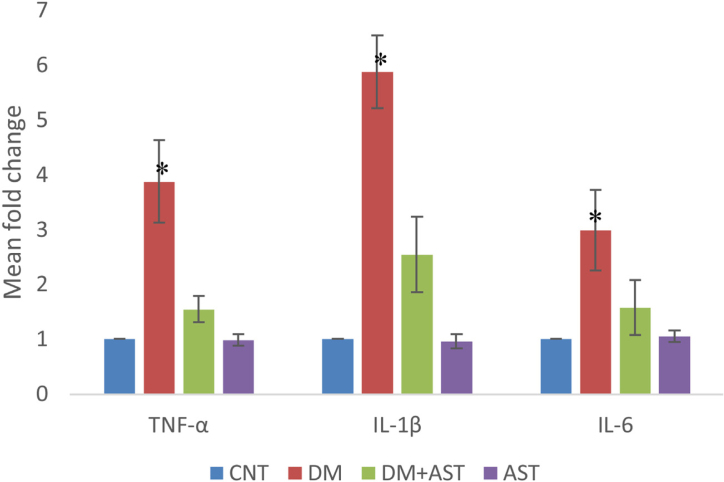
TNF-α, IL-1β and IL-6 values measured by real time PCR. TNF-α, IL-1β and IL-6 values were found to be significantly higher in the DM group when compared to other groups. (*p < 0.01 vs. other groups, ANOVA and posthoc Tukey test). CNT, Control; DM, Diabetes Mellitus; AST, Astaxanthin.

In cochlear tissue samples, real time PCR method was used for the analysis of TNF-α, IL-1β and IL-6 levels. It was observed that the levels of all three cytokines increased statistically (*p* < 0.01, Fig. 6) when the DM group was compared with the CNT group. In the DM + AST group, there was no increase in cytokine levels observed in the DM group (*p* < 0.01, Fig. 6).

## Discussion

The main objective of this study is to investigate the effects of AST, an antioxidant molecule, on hearing loss in diabetic rats. DM not only shortens the life expectancy with the chronic complications it causes, but also negatively affects the quality of life. Studies on this subject have shown that hearing problems are more common in diabetic individuals than in those who do not have this disease, and they start at an earlier age.^17^ It has been demonstrated that hearing loss in patients with DM is associated with glycemic control, and the risk is higher in people with poor control.^18^ The reason for the increased risk is the damage of high glucose levels to the auditory pathways and cochlear structures, especially the hair cells.^19^ Hyperglycemia leads to an increase in advanced glycation end-products and ROS in tissues. This causes oxidation and inflammation. As a result, these events damage the structures involved in hearing and cause deterioration in their functions.^8^, ^10^, ^20^ Tissues involved in hearing functions can secrete antioxidant enzymes, including GPx, SOD and CAT.^21^ GPx converts glutathione, which is involved in H_2_O_2_ detoxification, from its oxidized form to its reduced form. SOD catalyzes singlet oxygen and CAT catalyzes hydrogen peroxide. DM causes excessive ROS release in these tissues and a relative insufficiency of the mentioned antioxidant enzyme levels. As a result, ROS that cannot be adequately removed cause oxidative damage, lipid peroxidation, inflammation, and consequently cellular damage and hearing loss.^8^, ^9^, ^10^, ^11^

In this study, ABR and DPOAE hearing tests were applied to diabetic rats in order to evaluate their auditory functions. Both tests applied can successfully reveal the functional results of changes in the inner ear in humans and animals.^22^ ABR reflects the electrical activities of the auditory nerve and its brainstem connections and is mostly used to evaluate the neural aspect of hearing loss.^9^ DPOAE is a test that provides the means to evaluate the hearing loss due to outer hair cells damage in the cochlea with the measurement made in the outer ear canal.^6^, ^22^, ^23^ As a result of the measurements made with this test, there are studies reporting that DM causes hearing loss, especially against high-frequency sounds, and in some other studies, it has been said that hearing loss is valid for all frequencies. In this study, we observed significant deterioration in hearing functions in both ABR and DPOAE tests in the DM group. While no difference was observed between the groups at day 0 in the ABR test, ABR thresholds levels were gradually increasing with the effect of DM on days 30 and 60. In the DPOAE test, the 30th and 60th day measurements in the DM group were found to be significantly lower than the 0th day measurements. This occurred at both low and high frequencies. These results, in line with the literature, show that DM damages auditory pathways and cochlear structures. In the DM+AST group, hearing impairments observed in the DM group were not observed. In the DM+AST group, unlike the DM group, the measurements on Day 30 and day 60 were not different from the measurements on day 0. This is due to the protective effect of AST. AST was able to reverse the effect of DM in the auditory pathways and cochlea.

Although this study is the first in the literature to investigate the effects of AST on hearing loss due to DM, the effect of this molecule has been tried and found successful in many different conditions due to DM. Conditions in which AST is tried include retinopathy due to DM, cataracts, nephropathy, and neuropathy.^14^, ^24^, ^25^ The common point reached in these studies is that AST has beneficial effects by inhibiting oxidation and inflammation. AST is known as a powerful antioxidant with no side effects. AST quenching singlet oxygen, scavenging radicals prevent lipid peroxidation and suppress oxidative stress-related gene expression.^26^ AST inhibits the inflammatory response by suppressing the release of pro-inflammatory cytokines, including TNF-α, IL-1β and IL-6.^25^, ^27^

Lately, the protective effects of AST against cisplatin-induced autotoxicity have been reported in various studies. Gu and his colleagues reported alleviated cisplatin-induced autotoxicity and oxidation-related pathologies with AST.^28^ Two more researchers were shown similar results with AST against cisplatin-induced autotoxicity.^29^, ^30^ The researchers were pointed out that reduced oxidative stress and cellular damage with AST applications were responsible for the protective effects. One of these studies, Kınal, and colleagues,^29^ remarked that AST’s protective effects specifically against high-frequency hearing loss only at higher doses (40 mg/kg). Although the reason for autotoxicity was different, the present study results were in accordance with the literature data. The present study differed from Kınal and his colleagues with the applied AST dose (80 mg/kg), tested against high and low-frequency hearing loss. The results of this study reveal the protective effects of AST at higher doses.

In this study, while the levels of Gpx, SOD, CAT and MDA were analyzed in blood and cochlear tissue samples in order to reveal the antioxidant and anti-inflammatory effects of AST, the levels of pro-inflammatory cytokines such as TNF-α, IL-1β and IL-6 were only measured in cochlear tissue samples. MDA is a product of lipid peroxidation and is considered an important marker of oxidative stress.^6^, ^31^ In this study, while the MDA level increased in the DM group in blood and cochlear tissue samples, this increase was not observed in the DM+AST group. This result, which can be interpreted as being successful in preventing lipid peroxidation, is probably due to the fact that AST increases the level of antioxidant enzymes. Because Gpx, SOD and CAT levels were found to be significantly higher in the DM+AST group compared to DM. These enzymes are an important indicator of the body’s antioxidant capacity.^14^, ^32^ In this study, AST significantly increased the levels of all three enzymes. This ensures that the ROS scavenging ability remains at a high level, even in a situation such as DM that causes intense oxidation. AST also caused a decrease in the levels of pro-inflammatory cytokines such as TNF-α, IL-1β and IL-6, which were increased with DM in cochlear tissue. Considering that these cytokines increase oxidative damage and induce apoptosis, decreasing the levels of these substances by AST plays an important role in protecting auditory cells.

## Conclusion

As a result, when the data obtained is assessed, AST showed a protective effect against hearing disorders due to DM. AST demonstrated this beneficial effect by increasing antioxidant capacity and suppressing pro-inflammatory cytokines. Even though the effects of AST were investigated in a diabetic experimental animal model, there is a need to evaluate its therapeutic effects on humans. If proven successful in treating hearing-loss symptoms, AST can be suggested and prescribed to diabetic patients as an adjunct therapy with its antioxidant effects.

## Funding

This research did not receive any specific grant from funding agencies in the public, commercial, or not-for-profit sectors.

## Conflicts of interest

The authors declare no conflicts of interest.
